# Supplementary material for: Resolving the full spectrum of human genome variation using Linked-Reads
Source: Genome Res. 2019 Apr;29(4):635–45. doi: 10.1101/gr.234443.118 (PMC6442396; doi:10.1101/gr.234443.118)
Supplement: Supplemental Material [file supp_gr.234443.118_Supplemental_longranger-2.2.zip › longranger-2.2.tar.bz2!/lr2.2-public/lib/stan/cmdstan/stan/doc/api/html/autocovariance_8hpp_source.html]

Stan: src/stan/prob/autocovariance.hpp Source File


|  |  |
| --- | --- |
|  | Stan  2.5.0  probability, sampling & optimization |


- Main Page
- Related Pages
- Namespaces
- Classes
- Files

- File List
- File Members

All Classes Namespaces Files Functions Variables Typedefs Enumerations Enumerator Friends Macros Pages

- src
- stan
- prob

autocovariance.hpp

Go to the documentation of this file.

1 #ifndef STAN\_\_PROB\_\_AUTOCOVARIANCE\_HPP

2 #define STAN\_\_PROB\_\_AUTOCOVARIANCE\_HPP

3

4 #include <stan/prob/autocorrelation.hpp>

5 #include <stan/math/matrix/variance.hpp>

6

7 namespace stan {

8

9  namespace prob {

10

11

32  template <typename T>

33  void autocovariance(const std::vector<T>& y,

34  std::vector<T>& acov,

35  Eigen::FFT<T>& fft) {

36

37  stan::prob::autocorrelation(y, acov, fft);

38

39  T var = stan::math::variance(y) \* (y.size()-1) / y.size();

40  for (size\_t i = 0; i < y.size(); i++) {

41  acov[i] \*= var;

42  }

43  }

44

61  template <typename T>

62  void autocovariance(const std::vector<T>& y,

63  std::vector<T>& acov) {

64  Eigen::FFT<T> fft;

65  autocovariance(y,acov,fft);

66  }

67

68

69  }

70 }

71

72 #endif

autocorrelation.hpp

variance.hpp

stan::prob::autocovariance

void autocovariance(const std::vector< T > &y, std::vector< T > &acov, Eigen::FFT< T > &fft)

Write autocovariance estimates for every lag for the specified input sequence into the specified resu...

**Definition:** autocovariance.hpp:33

stan::math::variance

boost::math::tools::promote\_args< T >::type variance(const std::vector< T > &v)

Returns the sample variance (divide by length - 1) of the coefficients in the specified standard vect...

**Definition:** variance.hpp:24

stan::prob::autocorrelation

void autocorrelation(const std::vector< T > &y, std::vector< T > &ac, Eigen::FFT< T > &fft)

Write autocorrelation estimates for every lag for the specified input sequence into the specified res...

**Definition:** autocorrelation.hpp:56


---

[ Stan Home Page ]

*© 2011–2014,
Stan Development Team.*
